# Sexual risk taking behaviour: prevalence and associated factors. A population-based study of 22 000 Danish men

**DOI:** 10.1186/1471-2458-11-764

**Published:** 2011-10-05

**Authors:** Nina Buttmann, Ann Nielsen, Christian Munk, Kai L Liaw, Susanne K Kjaer

**Affiliations:** 1 Department of Viruses, Hormones and Cancer, Institute of Cancer Epidemiology, Danish Cancer Society, DK-2100 Copenhagen, Denmark; 2 Merck Research Laboratories, North Wales, Pennsylvania, USA; 3 Department of Obstetrics and Gynaecology, Rigshospitalet, University of Copenhagen, Denmark

## Abstract

**Background:**

Sexual habits and risky sexual behaviour strongly affect public health. Available data indicate that sexually transmitted infections are increasing in many EU countries. Changes in the epidemiology of sexually transmitted diseases across Europe are among other factors suggested to be driven by changes in sexual behaviour patterns. The purpose of our study is to assess the occurrence of risky behaviour in men aged 18-45 years from the general population. Furthermore, we aim to examine factors associated with risky sexual behaviour.

**Methods:**

A random sample of 33 000 Danish men (18-45 years) was selected from the general population. The participants (participation-rate: 71.0%) received a self-administered questionnaire which could be returned in a paper-based version or as a web-based questionnaire. Non-respondents were subsequently asked to participate in a telephone interview with the same questions as in the paper- or web-based questionnaire. We defined risky sexual behaviour as > 8 lifetime sexual partners, ≥2 new sexual partners in the past 6 months and intercourse with a commercial sex worker.

**Results:**

The Danish men reported having had sexual intercourse with a median of 8 female partners during their lifetime and 9.8% of the men have had ≥2 new sexual partners in the past 6 months. Sexual intercourse with a commercial sex worker was reported by 11.3% of the men. Furthermore, men reporting > 8 lifetime partners or ≥2 recent sex partners were more likely to have other risk taking behaviours such as early sexual debut, current smoking and regular binge drinking. A similar pattern was seen in men who had sex with a commercial sex worker.

**Conclusions:**

Our results show that a high proportion of Danish men have had sexual contact with a large number of partners, and risky sexual behaviour is closely related to other risk-taking behaviours such as smoking and binge drinking.

## Background

Available data indicate that sexually transmitted infections (STIs) are increasing in many EU countries [1]. Changes in the epidemiology of sexually transmitted diseases (STDs) across Europe are among other factors suggested to be driven by changes in sexual behaviour patterns [1]. This is supported by trends towards larger numbers of sexual partners, concurrent partnerships [2-4], increasing proportions of adolescents engaging in sexual intercourse at a young age [3,5,6] and inconsistent condom use with new partners which have been described previously [2,4]. Other determinants of sexual health and morbidity are socio-demographic factors [7,8] and risky lifestyle factors that co-occur with risky sexual behaviour [9-11].

The dynamics in STI incidences continue to create demands for updated general population estimates of variables such as rates of partner acquisition and contact with high risk groups for STIs as e.g. sex workers. National representative surveys on sexual behaviour have been conducted in the late 1980s and early 1990s in many European countries [12], also in Denmark [13]. The Nordic countries have at that time been considered 'liberal' in their attitudes toward sexuality, and more permissive in terms of sexual relationships, evident in larger numbers of partners over lifetime than most other European countries [12]. A long term evaluation of Swedish national sex surveys from 1989 to 2007 has recently drawn attention to increasing prevalences of multiple sexual partners and of casual sex without the use of a condom in the general population [14]. Increasing trends in a wide range of behaviours associated with increased risk of STI transmission, including numbers of heterosexual partners, concurrent partnership and payment for sex has also been described for the period from 1990 to 2000 in British national sex surveys [2].

The purpose of our study is to assess the occurrence of high-risk sexual behaviour in a large random sample of Danish men aged 18-45 years from the general population. Furthermore, we aim to examine potential associations between socio-demographic and lifestyle factors and risky sexual behaviour. To our knowledge, this is one of the largest studies concerning sexual behaviour in men from the general population.

## Methods

The study was conducted between November 2006 and June 2007. All residents of Denmark have a unique personal identification number (PIN), which contains information on gender and date of birth. With this number, all residents are registered in the computerized national civil personal register (CPR), with data on vital status, migration and current address. The PIN is used universally in the Danish society including all health registries. For scientific purposes, permission to use data from the CPR might be obtained from the Danish Data Protection Agency, provided that the information is treated in accordance with the Danish Act on the Processing of Personal Data [15]. According to this Danish law, the study was approved by the Danish Data Protection Agency. Using this system, we drew a random sample of men aged 18-45 years from the general male population with all male residents of Denmark 18-45 years having an equal chance of selection. All potential participants received written information about the study and had the opportunity to decline participation in the study either actively through e-mail, phone or by letter or passively, by not returning the questionnaire, men were not explicitly asked for their consent to participate. According to Danish law, no further ethical approval was necessary to conduct this study. Piloting of the questionnaire was done by an initial cognitive debriefing in a few persons to ensure that the questions were not difficult to understand and that the way the questions were interpreted by the participants was identical to what was intended from the investigators. Secondly the questionnaire was given to a larger sample of men in the age range included in the study, and they reported back to us about their reaction to the questions. Most of the questions have been used before in similar studies that we have been undertaking.

The study has been described in detail previously [16]. Briefly, 33 000 Danish men were invited to participate; of these, 487 were ineligible because they had moved before the initial contact, had emigrated, did not speak Danish, had severe mental disability or had died before contact, leaving 32 513 men as potential participants. A total of 23 080 (71.0%) men participated in the study, as 3835 refused and 5598 did not respond to the invitation. The response rate in relation to age was as follows: 71.9% response rate in men aged 36-45 years, 72.6% in men aged 26-35 years and 66.5% in the youngest men aged 18-25 years. In the present study, we excluded 12 men for whom there was a discrepancy between their personal identification number and reported age and 658 men who did not answer questions on their sexual debut or number of female sexual partners, leaving 22 410 men available for analysis.

All participants received a self-administered questionnaire by mail, which could be returned in a paper-based version or as a password-protected web-based questionnaire identical to the paper-based one. Non-respondents were subsequently contacted by phone and asked to participate in a telephone interview with the same questions as in the paper- or web-based questionnaire. The paper-based questionnaire was returned by 48.9% of the participants, 16.6% responded by means of the web-based questionnaire, and 34.5% participated in a subsequent telephone interview. The questionnaire included items on socio-demographic factors, lifestyle, history of STDs and on sexual habits.

### Statistical analysis

We estimated the prevalence of reported lifetime numbers of female sexual partners, new female sexual partners in the past 6 months and of ever having had sexual intercourse with a commercial sex worker according to age with 95% confidence intervals (CIs). In order to be able to compare with previous studies [2,17,18] the variable lifetime numbers of female sexual partners was categorized into 1, 2-4, 5-9 and 10+ in the descriptive analyses.

In the literature, risky sexual behaviour has been defined using a variety of variables such as "multiple" partners during lifetime (defined in the particular population), "multiple" partners within the last 6 or 12 months, and having paid for sex [2,14,19,20]. In the present study, risky sexual behaviour was considered to include 'having had more than 8 partners', which was the median number in our study population, 'having had two or more new female sexual partners in the past 6 months' and 'ever having had sexual intercourse with a commercial sex worker'.

Associations between the measures of risky sexual behaviour, socio-demographic and lifestyle factors were assessed for sexually active men by means of multiple logistic regression analysis (SAS version 9.1). The measure of association was the odds ratio (OR) with corresponding 95% CI. Mutually adjusted models, stratified by age were used to assess the magnitude of associations between the individual variables and risky sexual behaviour. Age was furthermore treated as a continuous variable in the models. Adjustment for mode of response (paper-based or web-based questionnaire or telephone interview) did not change the mutually adjusted estimates (data not shown), and this variable was therefore not included as a covariate in the final regression models.

## Results

### Distribution of sexual habits according to age

Table 1 shows the overall and age-specific distribution of the number of female sexual partners (lifetime and in the past 6 months) and any sexual intercourse with a commercial sex worker. A total of 4.1% of the men had never engaged in sexual intercourse with a female partner at the time of enrolment in the study, ranging from 15.6% (95% CI, 14.0-17.3) of men aged 18-20 years to 1.6% (95% CI, 1.2-1.9) of 41-45-year-old men. The lifetime number of partners ranged from four or fewer in the first quartile to ≥ 15 in the third quartile, with a median of eight female sexual partners (data not shown); 42.5% of the men reported having had sexual intercourse with 10 or more female partners during their lifetime, while 8.9% had had sexual intercourse with only one female partner (Table 1). The lifetime number of female sexual partners increased with age: 50.4% (95% CI, 49.0-51.9) of men aged 41-45 years, 40.6% (95% CI, 39.0-42.2) of men aged 26-30 years and 15.5% (95% CI, 13.9-17.2) of men aged 18-20 years reported having had 10 or more female partners during their lifetime.

**Table 1:** Distribution of sexual habits in the study population according to age at enrolment into the study

	**Age at entry in the study (years)**													
			
**Sexual habits**	**Total**		**18-20** **(n = 1,958)**		**21-25** **(n = 2,867)**		**26-30** **(n = 3,469)**		**31-35** **(n = 4,430)**		**36-40** **(n = 4,905)**		**41-45** **(n = 4,781)**	
						
	**N**	**%**	**%**	**(95% CI)**	**%**	**(95% CI)**	**%**	**(95% CI)**	**%**	**(95% CI)**	**%**	**(95% CI)**	**%**	**(95% CI)**
	
Total	22,410	100	8.7	(8.4-9.1)	12.8	(12.4-13.3)	15.5	(15.0-16.0)	19.8	(19.3-20.3)	21.9	(21.4-22.4)	21.3	(20.8-21.9)
														
**No sexual intercourse with a female partner**	916	4.1	15.6	(14.0-17.3)	7.6	(6.7-8.7)	3.5	(2.9-4.1)	2.4	(1.9-2.8)	1.9	(1.6-2.3)	1.6	(1.2-1.9)
														
**Lifetime number of female sexual partners**														
1	2,000	8.9	14.8	(13.3-16.5)	10.3	(9.2-11.5)	8.3	(7.4-9.3)	8.5	(7.7-9.4)	7.7	(7.0-8.5)	7.7	(7.0-8.5)
2-4	4,670	20.8	32.6	(30.6-34.8)	24.3	(22.8-25.9)	21.8	(20.4-23.2)	18.0	(16.9-19.2)	18.9	(17.8-20.0)	17.9	(16.8-19.0)
5-9	5,292	23.6	21.5	(19.7-23.4)	26.1	(24.5-27.7)	25.9	(24.4-27.4)	23.4	(22.1-24.6)	22.9	(21.7-24.1)	22.4	(21.2-23.6)
≥ 10	9,532	42.5	15.5	(13.9-17.2)	31.7	(30.0-33.4)	40.6	(39.0-42.2)	47.7	(46.3-49.2)	48.7	(47.3-50.1)	50.4	(49.0-51.9)
														
**Number of new female sexual partners in the past 6 months**														
0-1	19,172	85.6	62.1	(59.9-64.3)	70.4	(68.7-72.1)	84.6	(83.4-85.8)	90.5	(89.6-91.4)	92.7	(92.0-93.5)	93.0	(92.2-93.7)
≥ 2	2,186	9.8	22.1	(20.2-23.9)	21.5	(19.9-23.0)	11.3	(10.3-12.4)	6.6	(5.9-7.4)	4.8	(4.2-5.4)	4.6	(4.0-5.2)
														
**Sex with a commercial sex worker**														
Never	18,889	84.3	81.2	(79.4-82.9)	84.4	(83.1-85.8)	84.9	(83.7-86.1)	84.1	(83.0-85.2)	85.2	(84.2-86.2)	84.3	(83.2-85.3)
Ever	2,524	11.3	3.2	(2.5-4.1)	7.4	(6.5-8.5)	11.1	(10.1-12.2)	13.3	(12.3-14.4)	12.4	(11.5-13.4)	13.9	(12.9-14.9)

Because of missing values, the numbers might not add up to the total

The majority of the men (85.6%) had no or only one new sexual partner in the past 6 months, while 9.8% reported having had two or more new partners in that period. The proportion of men who had recently had two or more new sexual partners declined with age, from 22.1% (95% CI, 20.2-23.9) among men aged 18-20 years to 4.6% (95% CI, 4.0-5.2) of men aged 41-45 years (Table 1).

Sexual intercourse with a commercial sex worker was reported by 11.3% of the population. The prevalence of sexual intercourse with a commercial sex worker increased with age, from 3.2% (95% CI, 2.5-4.1) of the youngest men (18-20 years) to 13.9% (95% CI, 12.9-14.9) of men aged 41-45 years.

### Factors associated with risky sexual behaviour

Table 2 shows the results of the logistic regression analysis of the association between reporting more than eight female sexual partners during lifetime and two or more new female sexual partners in the past 6 months with selected socio-demographic and lifestyle factors. Overall, men reporting > 8 partners during lifetime were more likely to be older (OR_adj _= 1.08; 95% CI, 1.07-1.08 per year older), single (OR_adj _= 1.29; 95% CI, 1.20-1.39), have a high educational level (OR_adj _= 1.20; 95% CI, 1.10-1.32) and to live in the capital area (OR_adj _= 2.20; 95% CI, 2.00-2.41). With regard to lifestyle factors, we observed an increased likelihood of having had > 8 sex partners among current smokers (OR_adj _= 2.00; 95% CI, 1.87-2.14) and with increasing frequency of binge drinking (≥ 4/month; OR_adj _= 2.69; 95% CI, 2.35-3.07). Men who started sexual activity at a young age, defined as ≤ 14 years, were also more likely to report > 8 sexual partners (OR_adj _= 4.26; 95% CI, 3.86-4.72). In the age-stratified analyses associations between education and having had > 8 partners or residency and reporting > 8 partners were stronger in the older age groups than in youngest men. No major differences were seen between age groups in lifestyle factors (Table 2).

**Table 2:** Associations between > 8 female sex partners during lifetime and selected socio-demographic, lifestyle and sexual behaviour variables among sexually active men stratified by age (n = 21,494)

	> 8 female sex partners during lifetime							
	
	All ages		18-25 years		26-35 years		36-45 years	
				
	OR_adj_	(95% CI)	OR_adj_	(95% CI)	OR_adj_	(95% CI)	OR_adj_	(95% CI)
**Age at enrolment (years)**								
Linear (per year)	1.08	(1.07-1.08)	1.35	(1.30-1.40)	1.10	(1.08-1.12)	1.02	(1.00-1.03)
								
**Marital status**								
Married or cohabiting	1		1		1		1	
Single	1.29	(1.20-1.39)	1.71	(1.43-2.04)	1.43	(1.27-1.60)	1.52	(1.35-1.71)
								
**Educational level**								
≤ 9 years	1		1		1		1	
10-11 years	1.18	(1.08-1.29)	1.11	(0.89-1.39)	1.05	(0.89-1.25)	1.15	(1.03-1.30)
≥ 12 years	1.20	(1.10-1.32)	0.81	(0.65-1.02)	1.12	(0.94-1.33)	1.28	(1.12-1.45)
								
**Area of residence**								
Capital area	2.20	(2.00-2.41)	1.27	(1.02-1.59)	1.97	(1.72-2.26)	2.51	(2.11-2.98)
Capital suburbs	1.27	(1.15-1.41)	1.11	(0.87-1.41)	1.23	(1.02-1.49)	1.38	(1.20-1.59)
Large provincial cities	1.34	(1.23-1.46)	1.00	(0.82-1.22)	1.38	(1.21-1.59)	1.31	(1.15-1.50)
Rural areas	1		1		1		1	
								
**Smoking**								
Never	1		1		1		1	
Former	1.56	(1.44-1.70)	1.57	(1.19-2.06)	1.52	(1.32-1.75)	1.56	(1.39-1.74)
Current	2.00	(1.87-2.14)	1.81	(1.54-2.11)	2.23	(1.99-2.50)	1.83	(1.66-2.03)
								
**Alcohol consumption**								
Never binge drinking	1		1		1		1	
Never drinking	0.77	(0.52-1.15)	1.29	(0.59-2.85)	0.49	(0.21-1.12)	0.69	(0.39-1.23)
Binge drinking < 1/month	1.43	(1.28-1.59)	0.95	(0.63-1.45)	1.81	(1.48-2.21)	1.26	(1.10-1.45)
Binge drinking 1-3/month	2.19	(1.96-2.45)	1.54	(1.05-2.26)	2.92	(2.39-3.58)	2.05	(1.77-2.37)
Binge drinking ≥ 4/month	2.69	(2.35-3.07)	2.67	(1.80-3.96)	4.16	(3.24-5.34)	2.30	(1.88-2.81)
								
**Age at first intercourse with a female partner (years)**								
≥ 15	1		1		1		1	
≤ 14	4.26	(3.86-4.72)	5.60	(4.61-6.81)	4.05	(3.42-4.79)	4.49	(3.76-5.36)

All ORs are mutually adjusted for all variables in the table and for age as a continuous variable

We observed that overall, participants reporting two or more new recent partners were more likely to be single (OR_adj _= 20.34; 95% CI, 17.44-23.72) and to live in the capital area (OR_adj _= 1.44; 95% CI, 1.25-1.66) (Table 3). Current smoking (OR_adj _= 1.21; 95% CI, 1.08-1.36) and regular binge drinking (OR_adj _= 3.23; 95% CI, 2.52-4.14) were also associated with an increased risk of having had two or more new sexual partners within the past 6 months. In contrast, we found no association with educational level. As observed for the lifetime number of partners, men who reported having had ≥ 2 new recent sexual partners were more likely to have had an early sexual debut (OR_adj _= 2.25; 95% CI, 1.96-2.58). The estimates stratified by age pointed generally in the same direction. However, current smoking was only significantly associated with having had ≥ 2 recent partners in the youngest age group, 18-25 years (OR_adj _= 1.40; 95% CI, 1.18-1.65) (Table 3), and the strongest association with marital status was observed in the oldest men (OR_adj _= 26.73; 95% CI, 20.59-34.70).

**Table 3:** Associations between ≥ 2 new female sexual partners in the past 6 months and selected socio-demographic, lifestyle and sexual behaviour variables among sexually active men stratified by age (n = 21,494)

	≥ 2 new female sexual partners in the past 6 months							
	
	All ages		18-25 years		26-35 years		36-45 years	
				
	OR_adj_	(95% CI)	OR_adj_	(95% CI)	OR_adj_	(95% CI)	OR_adj_	(95% CI)
**Age at enrolment (years)**								
Linear (per year)	0.99	(0.98-0.99)	1.05	(1.02-1.10)	0.97	(0.94-1.00)	0.98	(0.95-1.02)
								
**Marital status**								
Married or cohabiting	1		1		1		1	
Single	20.34	(17.44-23.72)	11.89	(8.79-16.09)	23.20	(18.21-29.57)	26.73	(20.59-34.70)
								
**Educational level**								
≤ 9 years	1		1		1		1	
10-11 years	1.14	(0.98-1.33)	1.07	(0.84-1.36)	1.08	(0.79-1.46)	1.22	(0.91-1.63)
≥ 12 years	0.99	(0.84-1.16)	0.85	(0.67-1.07)	0.94	(0.68-1.29)	1.28	(0.93-1.76)
								
**Area of residence**								
Capital area	1.44	(1.25-1.66)	1.41	(1.11-1.79)	1.04	(0.83-1.32)	1.65	(1.23-2.21)
Capital suburbs	1.09	(0.91-1.30)	1.14	(0.89-1.47)	0.86	(0.59-1.25)	1.25	(0.89-1.77)
Large provincial cities	1.16	(1.00-1.33)	1.18	(0.96-1.45)	0.95	(0.74-1.22)	1.06	(0.76-1.49)
Rural areas	1		1		1		1	
								
**Smoking**								
Never	1		1		1		1	
Former	0.91	(0.76-1.09)	1.19	(0.87-1.61)	0.80	(0.59-1.10)	0.72	(0.52-0.99)
Current	1.21	(1.08-1.36)	1.40	(1.18-1.65)	1.11	(0.91-1.36)	0.92	(0.72-1.16)
								
**Alcohol consumption**								
Never binge drinking	1		1		1		1	
Never drinking	0.81	(0.41-1.60)	0.92	(0.38-2.25)	0.25	(0.03-1.97)	0.79	(0.18-3.61)
Binge drinking < 1/month	1.06	(0.83-1.37)	0.76	(0.47-1.24)	1.19	(0.75-1.90)	1.21	(0.81-1.79)
Binge drinking 1-3/month	2.12	(1.67-2.69)	1.48	(0.96-2.27)	2.63	(1.69-4.11)	2.49	(1.69-3.68)
Binge drinking ≥ 4/month	3.23	(2.52-4.14)	2.57	(1.67-3.96)	4.65	(2.90-7.45)	2.46	(1.58-3.82)
								
**Age at first intercourse with a female partner (years)**								
≥ 15	1		1		1		1	
≤ 14	2.25	(1.96-2.58)	2.35	(1.91-2.88)	1.99	(1.53-2.60)	2.46	(1.86-3.26)

All ORs are mutually adjusted for all variables in the table and for age as a continuous variable

The associations with having had sexual intercourse with a commercial sex worker are shown in Table 4. The risk for reporting having had sexual intercourse with a commercial sex worker increased with age (OR_adj _= 1.05; 95% CI, 1.04-1.05 per year older). Being single and living in the capital area also increased the likelihood of such sexual contact, as did current smoking (OR_adj _= 1.28; 95% CI, 1.16-1.42) in the overall analysis. We also observed significant associations with early age at first intercourse (OR_adj _= 1.19; 95% CI, 1.06-1.34), multiple sexual partners during lifetime (OR_adj _= 12.81; 95% CI, 8.50-19.31 for ≥ 10 vs. 1 sexual partner) and multiple new recent sexual partners (OR_adj _= 1.88; 95% CI, 1.62-2.18 for ≥ 2 vs. 0 new partners). In contrast, no strong overall association was seen between having paid for sex and binge drinking frequency. The age-stratified estimates were largely following the same pattern across age groups. The association with educational level showed a pattern of a decreasing risk with increasing level of education, and the association with lifetime number of partners was largely driven by men in the oldest age group (men 36-45 years: OR_adj _= 22.19; 95% CI, 9.87-49.87 for ≥ 10 vs. 1 sexual partner).

**Table 4:** Associations between ever having had sexual intercourse with a commercial sex worker and selected socio-demographic, lifestyle and sexual behaviour variables among sexually active men stratified by age (n = 21,494)

	Sexual intercourse with a commercial sex worker							
	
	All ages		18-25 years		26-35 years		36-45 years	
				
	OR_adj_	(95% CI)	OR_adj_	(95% CI)	OR_adj_	(95% CI)	OR_adj_	(95% CI)
**Age at enrolment (years)**								
Linear (per year)	1.05	(1.04-1.05)	1.21	(1.13-1.30)	1.05	(1.02-1.07)	1.00	(0.98-1.03)
								
**Marital status**								
Married or cohabiting	1		1		1		1	
Single	1.60	(1.43-1.80)	2.32	(1.59-3.38)	1.47	(1.22-1.76)	1.76	(1.49-2.08)
								
**Educational level**								
≤ 9 years	1		1		1		1	
10-11 years	0.99	(0.87-1.12)	0.84	(0.58-1.22)	0.95	(0.76-1.20)	0.95	(0.80-1.13)
≥ 12 years	0.85	(0.74-0.97)	0.71	(0.49-1.03)	0.76	(0.60-0.97)	0.89	(0.73-1.07)
								
**Area of residence**								
Capital area	1.32	(1.16-1.50)	1.33	(0.92-1.92)	1.06	(0.87-1.28)	1.40	(1.15-1.71)
Capital suburbs	1.19	(1.02-1.38)	1.51	(1.01-2.25)	1.33	(1.03-1.73)	1.10	(0.89-1.35)
Large provincial cities	1.05	(0.93-1.20)	0.91	(0.63-1.31)	1.09	(0.89-1.33)	0.99	(0.81-1.21)
Rural areas	1		1		1		1	
								
**Smoking**								
Never	1		1		1		1	
Former	1.07	(0.94-1.21)	2.10	(1.35-3.26)	1.11	(0.90-1.37)	0.96	(0.81-1.14)
Current	1.28	(1.16-1.42)	1.70	(1.27-2.27)	1.23	(1.04-1.44)	1.22	(1.06-1.42)
								
**Alcohol consumption**								
Never binge drinking	1		1		1		1	
Never drinking	0.78	(0.39-1.54)	1.33	(0.32-5.51)	0.98	(0.33-2.93)	0.48	(0.14-1.61)
Binge drinking < 1/month	0.94	(0.80-1.12)	1.63	(0.73-3.62)	0.81	(0.61-1.10)	0.91	(0.73-1.12)
Binge drinking 1-3/month	0.95	(0.80-1.12)	1.16	(0.54-2.46)	0.89	(0.66-1.19)	0.91	(0.72-1.13)
Binge drinking ≥ 4/month	1.15	(0.95-1.40)	1.41	(0.66-3.02)	1.03	(0.73-1.44)	1.56	(1.20-2.02)
								
**Age at first intercourse with a female partner (years)**								
≥ 15	1		1		1		1	
≤ 14	1.19	(1.06-1.34)	1.31	(0.95-1.80)	1.12	(0.93-1.36)	1.28	(1.08-1.53)
								
**Lifetime number of sexual partners**								
1	1		1		1		1	
2-4	2.55	(1.65-3.93)	0.82	(0.39-1.73)	3.23	(1.54-6.79)	4.54	(1.96-10.50)
5-9	5.18	(3.41-7.86)	1.81	(0.90-3.68)	6.78	(3.31-13.91)	8.08	(3.56-18.37)
≥ 10	12.81	(8.50-19.31)	3.57	(1.75-7.29)	14.52	(7.14-29.53)	22.19	(9.87-49.87)
								
**Number of new sex partners in past 6 months**								
0	1		1		1		1	
1	1.36	(1.21-1.53)	1.62	(1.13-2.31)	1.31	(1.09-1.58)	1.34	(1.13-1.60)
≥ 2	1.88	(1.62-2.18)	1.54	(1.04-2.27)	2.05	(1.61-2.60)	2.63	(2.07-3.34)

All ORs are mutually adjusted for all variables in the table and for age as a continuous variable

## Discussion

In this large population-based study of more than 22 000 Danish men aged 18-45 years, we found that more than 40% of the study population had had sexual intercourse with 10 or more female partners, with a median lifetime number of 8 sexual partners and nearly 10% reported having had two or more new sexual partners in the past 6 months. Other key findings include the close association between risky sexual behaviours and other risk-taking behaviours, such as current smoking and regular binge drinking.

The highest proportions of men with 10 or more partners during lifetime are amongst the oldest age group in our study. This increasing trend in numbers of partners during lifetime in successive birth cohorts has also been seen in a previous Nordic study [17] but contrasting previous results from a study conducted in Denmark in 1989 by Melbye and Biggar [13]. The authors showed highest numbers of recent and sexual partners during lifetime among the 30-34 year old men. They relate their findings to the liberalization of sexual attitudes that had an impact on the generation becoming sexually active in the 1970s. In our study population of men born between 1961 and 1988, a general liberal attitude towards sexuality had been established and might explain the linear increasing trend that we observed in numbers of partners during lifetime. In addition, the older birth cohorts also had a longer time to accrue new sexual partners.

Slightly lower median and total numbers of partners during lifetime and of recent partners have been described in two other recent nationwide sex surveys in Europe [2,18]. Considering large numbers of partners as risk factor for negative sexual health outcomes, Danish men seem to place themselves as well as their respective partners at comparably high risks for e.g. STDs.

In our study population, a total of 11.3% of the men had ever had sex with a commercial sex worker. This is in line with a recent study from Norway reporting that 12.9% of Norwegian men had paid for sex [19]. The prevalence of having sex with a commercial sex worker seems to have been rather stable in the last 10-15 years as our results are similar to an earlier Danish study from 1989 of 1,466 men aged 18-59 years [13]. In contrast, British national surveys reported a 2-fold increase from 1990 to 2000 [2,21]. Other recent European general population based studies on the prevalence of ever having paid for sex in men aged 18-49 years range from substantially lower (4.4% in Slovenia) to substantially higher prevalence (25.4% in Spain), compared with our results [18,22].

We found that in our male study population, > 8 sexual partners, ≥ 2 recent new sexual partners and sexual contact with a commercial sex worker, which we considered as risky sexual behaviour, is strongly associated with other risk-taking behaviours, such as current smoking and regular binge drinking. This association is evident in all age groups. Our findings in men from the general population support other studies that relied on selected study samples such as high school students where risky sexual behaviour increased e.g. with substance use [10,23,24]. Furthermore, we find sexual risk behaviour to be associated with early age at first intercourse. The association between risky sexual behaviour and other risk taking behaviours observed in our study as well as in other studies, supports the occurrence of a clustering of risk taking behaviours [25].

The major strengths of this study include the high response rate and the large study population that covers a broad age spectrum in men. A response rate of more than 70% among men has rarely been achieved in general population samples. The high response rate reduces the risk for selection bias and makes our results potentially more generalizable to the male Danish population of the same age groups. The large number of participants implies stable estimates and statistical power. A potential limitation includes bias introduced by selective participation as it has been suggested that participants in national sexuality surveys might have a higher socioeconomic status and might be better educated than non-participants [26]. Comparing socio-demographic data from the present study to the statistical database "Statistics Denmark", which contains detailed statistical information on the Danish population demonstrates a similar distribution of the variables age, level of schooling and marital status [27]. The questionnaire only addressed heterosexual behaviour; our results can therefore not necessarily be extrapolated to men who have sex with men (MSM). It is estimated that MSM in Denmark constitute 2.5% of the adult male population [28]. Inherent limitations of surveys on sensitive topics might also have affected our results. A major concern in studies on sexual behaviour is validity of the collected information. People with stigmatised or risky behaviours may be more likely not to participate in the study or under-report risky behaviour [29] however, this bias probably does not seriously compromise population estimates [30]. A generally liberal attitude towards sex in Denmark might furthermore reduce this source of bias. We also attempted to reduce bias and strengthen the validity of the responses by phrasing the questions in our survey in a non-judgemental and formal way, by removing all individual identifiable information from the questionnaires (i.e. they were only marked with a random study number), and by using password protected log-in for the web-based questionnaire to assure confidentiality.

## Conclusions

In conclusion, our results show that a high proportion of Danish men have sexual contacts with a large number of partners. Our results underscore the importance of effectively targeting both younger and older individuals to reappraise risk perception and promote healthy sexual behavior. Scientific research and continuous monitoring of behavioural changes will advance our understanding on the effectiveness and impact of population based prevention programmes. We have also shown that risky sexual behaviour is closely related to other risk-taking behaviours, such as smoking and binge drinking. This accumulation of risky behaviours indicates the need for a wider perception of risk taking behaviour in prevention programs concerning men's health.

## Competing interests

NB and AN declare that they have no competing interests. KLL is a current employee of Merck & Co. Inc. CM has received support for travel and conference participation from Merck & Co. Inc. SKK has received travel and institutional research grants from Merck & Co. Inc. and Sanofi Pasteur MSD.

## Authors' contributions

NB carried out the statistical analyses, interpreted the data, drafted and finalized the manuscript. AN has been involved in drafting the manuscript and revising it critically for important intellectual content. KLL has been involved in revising the manuscript critically for important intellectual content. CM has made substantial contributions to conception, design and collection of data and has been involved in revising the manuscript critically for important intellectual content. SKK was leading the conception, design, and collection of data. She interpreted the data and has been involved in drafting the manuscript and revising it critically for important intellectual content and has given final approval of the version to be published.

All authors read and approved the final manuscript.

## Pre-publication history

The pre-publication history for this paper can be accessed here:

http://www.biomedcentral.com/1471-2458/11/764/prepub
